# Improve thermostability of *Bacillus* sp. TS chitosanase through structure-based alignment

**DOI:** 10.1038/s41598-021-95369-w

**Published:** 2021-08-04

**Authors:** Zhanping Zhou, Xiao Wang

**Affiliations:** 1 Tianjin Sinonocy Biological Technology Co. Ltd., Tianjin, 300308 China; 2 Nanfang College of Sun Yat-Sen University, Guangzhou, 510970 China

**Keywords:** Protein design, Metabolic engineering

## Abstract

Chitosanases can catalyze the release of chitooligosaccharides which have a number of medical applications. Therefore, Chitosanases are good candidates for large-scale enzymatic synthesis due to their favorable thermostability properties and high catalytic efficiency. To further improve the thermostability of a chitosanase from *Bacillus* sp. TS, which has a half-life of 5.32 min, we mutated specific serine residues that we identified as potentially relevant through structure comparison with thermophilic CelA from *Clostridium thermocellum*. Out of a total of 15 mutants, three, namely S265G, S276A, and S347G, show higher thermostability. Their half-lives at 60 °C were calculated as 34.57 min, 36.79 min and 7.2 min. The *K*_m_ values of S265G, S276A and S347G mutants show substrate binding ability comparable to that of the wild-type enzyme, while the S265G mutant displays a significant decrease of enzymatic activities. Additionally, we studied the synergistic effects of combined mutations, observing that all double mutants and the triple mutant are more stable than the wild-type enzyme and single mutants. Finally, we investigated the mechanisms which might give a reasonable explanation for the improved thermostability via comparative analysis of the resulting 3D structures.

## Introduction

Chitosanase (EC 3.2.1.132) is a kind of enzyme that can degrade chitosan and produce chitooligosaccharides^1^, which are mainly composed of D-glucosamine and occasionally incorporation of *N*-acetylglucosamine. The chitooligosaccharides exhibit a wide range of interesting biological activities. A number of potential applications of chitooligosaccharides have been suggested in medical contexts, including their use as inhibitors of tumor growth and metastasis, anti-inflammation therapeutics against asthma, facilitators of bone-tissue formation, wound healing accelerators and antibacterial, antifungal, and anti-malaria agents^2–4^.

Degradation of chitosan requires cleavage of its β-1,4-glycosidic link between monomers. The thermostability of chitosanases is key to their industrial applicability; a thermostable chitosanase is beneficial for the bioconversion of chitosan because of the improved reaction rate, reduced substrate viscosity and the minimized risk of microbial contamination. In addition, it allows enzyme feeding to be decreased, which can cut down the costs and increases the flexibility of manufacture process during industrial scale-up.

Previously, we cloned and over-expressed a chitosanase gene of *Bacillus* sp. TS in *E. coli* and the enzymatic properties were also characterized^5^. This recombinant CsnTS belonged to the GH-8 family and displayed a maximal activity at 60 °C and pH 5.0 with (GlcN)_3–6_ as its main hydrolytic product^5^. In principle, these properties would make CsnTS a suitable candidate for industrial applications. However, CsnTS becomes unstable at temperatures above 60 °C. Therefore, improving the thermostability of CsnTS is essential to make this system useful for industrial products, such as large-scale production of chitooligosaccharides.

Increasing the thermostability of proteins generally requires mutations to the peptide chain of amino acid residuals composing the protein^6^. Direct evolution followed by candidates screening has been commonly used to improve an enzyme’s thermostability and catalytic properties^7,8^. Other than these methods, structure-based rational design has emerged as yet another useful method^9–13^. For instance, comparison of an enzyme’s amino acid sequence or structure with that of thermostable and highly homologous counterparts has given important clues and led to several novel thermostable enzymes^14–18^. The thermostability of proteins was controlled by various factors^19,20^, including electrostatic interactions, amino acid charges, aromatic interactions, and compactness. Moreover, statistical analyses have shown that serine residues appear in thermophilic proteins only with low frequency and that glycine and alanine are beneficial for protein stability^21,22^.

Accordingly, in this study, we employ structure and sequence comparison of CsnTS with the thermophilic homologous protein (CelA) from *Clostridium thermocellum*^23^, and introduce mutations of serine residues^24^ to improve the thermostability of CsnTS. A total of 15 variants with single-site mutation were screened, from which mutants S265G, S276A and S347G exhibited a higher thermostability than the wild-type CsnTS. We examine the biochemical properties of these mutants and elucidate potential reasons for their improved thermostability. Furthermore, we show that combinations of selected single-site mutations lead to double mutants and a triple mutant that are even more stable than the single-site mutants. Our intramolecular interaction analysis shows that the number of interactions among residues decreases in the triple mutant S265G/S276A/S347G compared to the wild-type CsnTS. Our results do not only provide thermostable chitosanases for industrial applications but also demonstrate a useful approach for rational protein engineering.

## Materials and methods

### General

The chitosanase gene of *Bacillus* sp. TS (GenBank AC No.: KU363821) was obtained from the plasmid produced in our previous study^5,25^. The *Escherichia coli* Trans10 and BL21 (DE3) strains were commercial products of TransGen Biotech (Beijing, China) and the strains were cultured in LB medium at 37 °C with kanamycine (50 μg/mL). Chitosan (degree of deacetylation > 90%) was also commercial products (Weihai Disha Marine Biological Products Co., LTD).

### Computer-aided modeling of CsnTS and the mutants

The theoretical structure of CsnTS and its mutants were constructed using Swiss-Model (http://swissmodel.expasy.org/), an online protein structure homology modeling server based on the reported structure of the chitosanase Chok (1V5C) of *Bacillus* sp. K17 as template^26^. CsnTS displayed highly homologous sequence (98% identity) as that of Chok. The final models herein displayed an excellent geometry without any disallowed residuals. Structure alignment was performed with the software BIOVIA DS (Accelrys, USA). Intramolecular interactions in both wild-type and mutant proteins were also calculated with DS software.

### Site-directed mutagenesis

The previously reported CsnTS gene was cloned into pET29a (+) vector between the unique restriction sites *Nde*I and *Xho*I to make the expression plasmid. We used this plasmid as template in site-directed mutagenesis PCR. PCR-amplification was performed to get the mutagenesis with the Fast Mutagenesis System kit from Transgen Biotech Co. LTD using primers that are listed in Table 1. The PCR products were digested by DMT enzyme for 1 h, then transformed into the competent *E. coli* supplied in the kit. For multiple site mutants, the constructed plasmids in the first PCR mutagenesis were used as templates. The correctness of all plasmids was confirmed via DNA sequencing. Finally, the sequence confirmed plasmids would be over-expressed in *E. coli* BL21 (DE3).

**Table 1:** List of primers.

Name	Sequence	Description
S51G_F	TTGAAAAATGATTTA**GGT**TCTTTACCT	S51G
S51G_R	CCACCAGGTAAAGA**ACC**TAAATCATTT	S51G
S52G_F	AAAAATGATTTATCT**GGT**TTACCTGGT	S52G
S52G_R	TAACCACCAGGTAA**ACC**AGATAAATCA	S52G
S112G_F	AACTTTTAAA**GGC**TCTCAAAATCCTA	S112G
S112G_R	TTTTGAGA**GCC**TTTAAAAGTTCTTGC	S112G
S126G_F	TGGGTTGTCGCAGAT**GGT**AAAAAAGC	S126G
S126G_R	CTTGTGCTTTTTT**ACC**ATCTGCGACA	S126G
S135A_F	CAAGGTCATTTTGAT**GCT**GCTACTGA	S135A
S135A_R	CCCCATCAGTAGC**AGC**ATCAAAATGA	S135A
S206A_F	TCTGATTGGATGATG**GCA**CACCTTAG	S206A
S206A_R	ATGCTCTAAGGTG**TGC**CATCATCCAA	S206A
S246G_F	AGGACTTATT**GGA**GATTTTGTTGTAAA	S246G
S246G_R	AACAAAATC**TCC**AATAAGTCCTGTATT	S246G
S265G_F	CTTAAATGAG**GGA**GAATATACAAATGC	S265G
S265G_R	TGTATATTC**TCC**CTCATTTAAGAAGTC	S265G
S276A_F	TATTATTATAATGCT**GCT**CGAGTACCT	S276A
S276A_R	CTTAAAGGTACTCG**AGC**AGCATTATAA	S276A
S276G_F	TATTATTATAATGCT**GGT**CGAGTACCT	S276G
S276G_R	CTTAAAGGTACTCG**ACC**AGCATTATAA	S276G
S329A_F	GGATCCAATATTGGT**GCT**TATCCAACT	S329A
S329A_R	ACACCAGTTGGATA**AGC**ACCAATATTG	S329A
S337G_F	ACTGGTGTATTCGTT**GGG**CCATTTATT	S337G
S337G_R	GCAGCAATAAATGG**CCC**AACGAATACA	S337G
S347G_F	TATAACAAAT**GGC**AATAATCAAAAGT	S347G
S347G_R	TGATTATT**GCC**ATTTGTTATACTTGC	S347G
S355G_F	GTGGGTAAAT**GGC**GGTTGGGATTGG	S355G
S355G_R	TCCCAACC**GCC**ATTTACCCACTTTTG	S355G
S369G_F	AGGCTATTTT**GGT**GATAGTTATAATT	S369G
S369G_R	TAACTATC**ACC**AAAATAGCCTTCTCT	S369G

Mutated nucleotides are in bold.

### Enzymatic activity assay

Chitosanase activity was evaluated by the method described below. 1 g of chitosan was dissolved in reaction buffer (100 mM sodium acetate, pH 5.0) to make substrate solution. Chitosanase was added to the substrate solution to start the hydrolysis reaction. The hydrolysis of chitosan reaction was carried out at 50 °C for a quarter. The released reducing sugar was quantified by the dinitrosalicylic acid method^5^. In one minutes, the amount of chitosanase required to release 1 μmol reducing sugar was defined as 1 unit enzyme. *D*-glucosamine was used to make calibration curve. The reaction mixture without enzyme was measured in a control experiment.

### Screening for mutants with increased thermostability

All plasmids to expression proteins mutants were transformed into expression host and spread on LB plates with 25 µg mL^−1^ of kanamycin. Fresh monocolonies were picked out and cultured in 50 mL of LB medium with according antibiotics at 37 °C with rotatory shaker. The induction was started by putting 0.1 mM IPTG at the culture OD_600_ of 0.5–0.8. The cultures were centrifuged at 3000*g* for 15 min. The cell pellets were suspended with HT buffer that contains 10 mM MgCl_2_ and 100 mM KCl dissolved in 50 mM HEPES–KOH (pH 7.6) and then disrupted by sonication. The crude enzyme solutions were obtained by centrifugation at 20,000*g* for 45 min at 4 °C. Crude enzyme solutions were then incubated at 55 °C for 30 min. 100 µL of each sample was used for enzymatic activity measurements.

### Protein expression and purification

The protein expression was similar to the process described above but scale up the culture volume to 500 mL. Crude enzyme solutions were also obtained by sonification of *E. coli* pellets.

The over-expressed proteins were purified by NTA-Ni affinity chromatography resin using GE commercial His-Trap FF columns as reported previously^5,25^. The purified proteins were stored in a HT buffer at − 20 °C. The protein purity was determined to 98% by SDS-PAGE (12% gel), and the protein concentrations were evaluated using a Bradford protein assay kit.

### Biochemical characteristics of the enzymes

The thermostabilities of purified wild-type CsnTS and its variants were determined by pre-incubating the proteins at 60 °C for various periods and then measuring the residual enzymatic activities under the standard assay conditions. The enzyme’s half-life (*t*_1/2_) was used to evaluate its thermostability.

To determine the optimal temperature, enzymatic activity was investigated at temperatures from 40 to 80 °C with 5 °C increments. For each protein, the maximal enzymatic activity under optimal temperature was defined to 100%, relative activity was the ratio of the enzymatic activity at an appointed temperature to the maximal activity.

The optimal pH was also investigated over a pH range of 3.5–7.5. pH 3.5–5.8 range was achieved using buffer with 100 mM sodium acetate and pH 6.0–7.5 range was achieved using 100 mM sodium phosphate buffer. All the reactions were performed at 50 °C. Like calculation in optimal temperature, the maximal activity at the optimal pH was defined to 100%, then relative activity was the ratio of the enzyme activity to the maximal activity at an indicated pH.

The reaction to obtain kinetic parameters of the wild-type CsnTS and its variants were carried out under standard enzymatic assay conditions. In the reactions, the concentration of enzyme was 0.5 U/mL enzymes, while chitosan concentrations ranged from 0.1 to 10 mg/mL. The experiment results were analyzed by fitting data sets to the enzyme kinetic equation of the Michaelis–Menten model using GraphPad Prism.

### Thermal unfolding of the enzymes

The *T*_m_ values of proteins was determined using Differential Scanning Calorimetry (DSC) measurements with a Nano DSC scanning microcalorimeter (Model 5100, Calorimetry Science Corporation, Utah, USA). The concentration of each protein sample was 0.2 mg/mL in HT buffer. And increased the temperature from 40 to 80 °C at a heating rate of 1 °C per min, with pressure set to 3.0 atm. The data were analyzed using the self-contained software NanoAnalyze.

## Results

### Identification of mutants with increased thermostability

**Figure 1:**
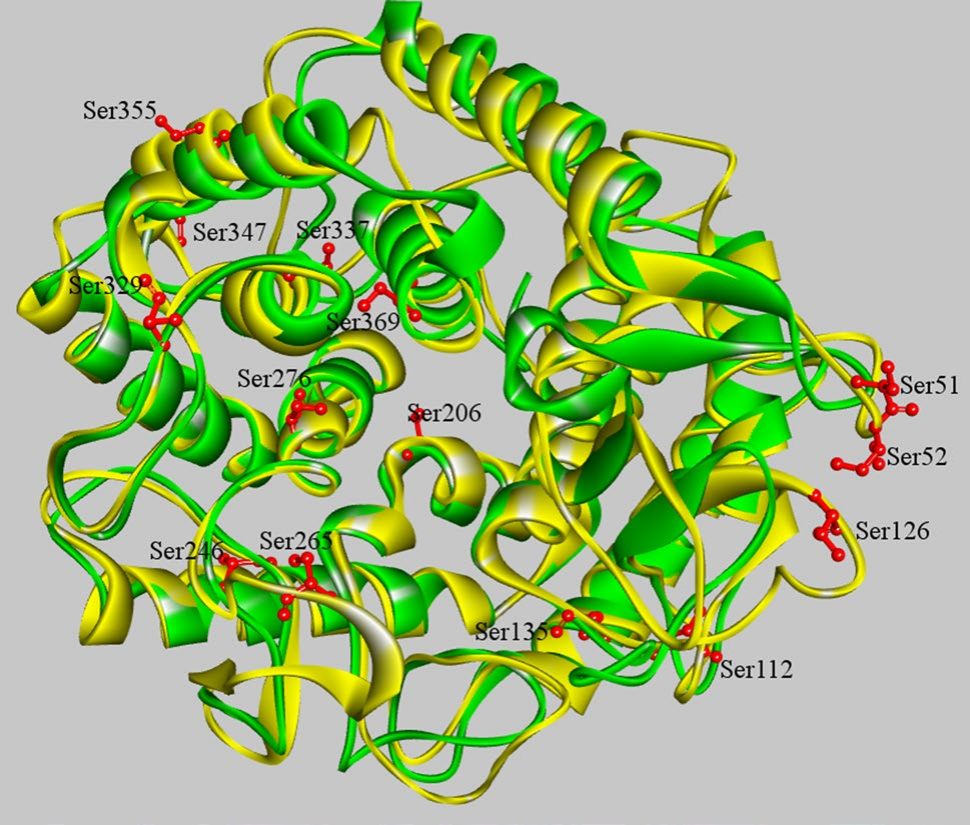
Comparisons of model structure of CsnTS and crystal structure of CelA. The model structure of CsnTS was constructed using the crystal structure of Chok (1V5C) as a template. The CsnTS was colored by yellow and the CelA was colored by green, respectively. The mutation sites in CsnTS were also displayed and represented by stick.

**Figure 2:**
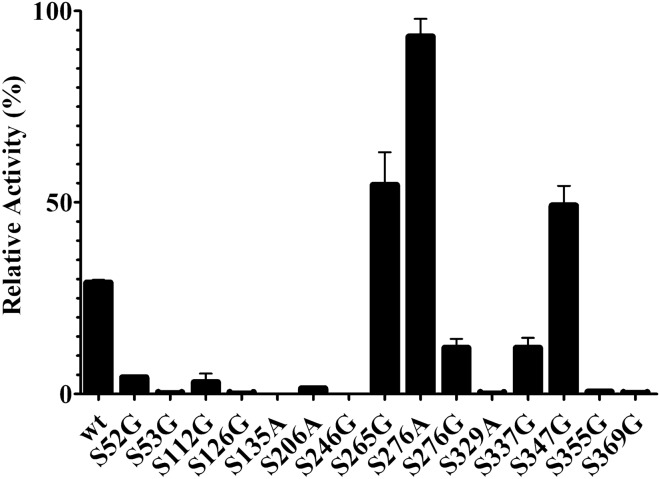
Screening of beneficial mutations. Residual activity of the wild-type and mutant proteins after pre-incubation at 60 °C for various times.

*Clostridium thermocellum* is a thermophilic bacterium. Its protein CelA possesses an optimal enzymatic temperature of 75 °C^23,27^. The structure alignment of CsnTS and CelA from *Clostridium thermocellum* was performed in order to determine potential target residues for subsequent engineering of mutations (Fig. 1). 15 serine residues were selected and mutated to alanine or glycine. The mutants were overexpressed in *E. coli* BL21 (DE3) strain. The harvested cells were crushed by ultra-sonication and lysates were used for activity measurement. The activities of the wild-type CsnTS and 15 mutations were evaluated with or without heat treatment. The retention of enzymatic activity was measured after incubating the samples for 30 min at 55 °C. The wild-type CsnTS retained approximately 20% of its activity after heating treatment. As shown in Fig. 2, the preliminary screening results showed that mutants S265G, S276A and S347G displayed enhanced thermostability. Consequently, these three mutants were purified for further studies.

### Thermostability of CsnTS variants

**Figure 3:**
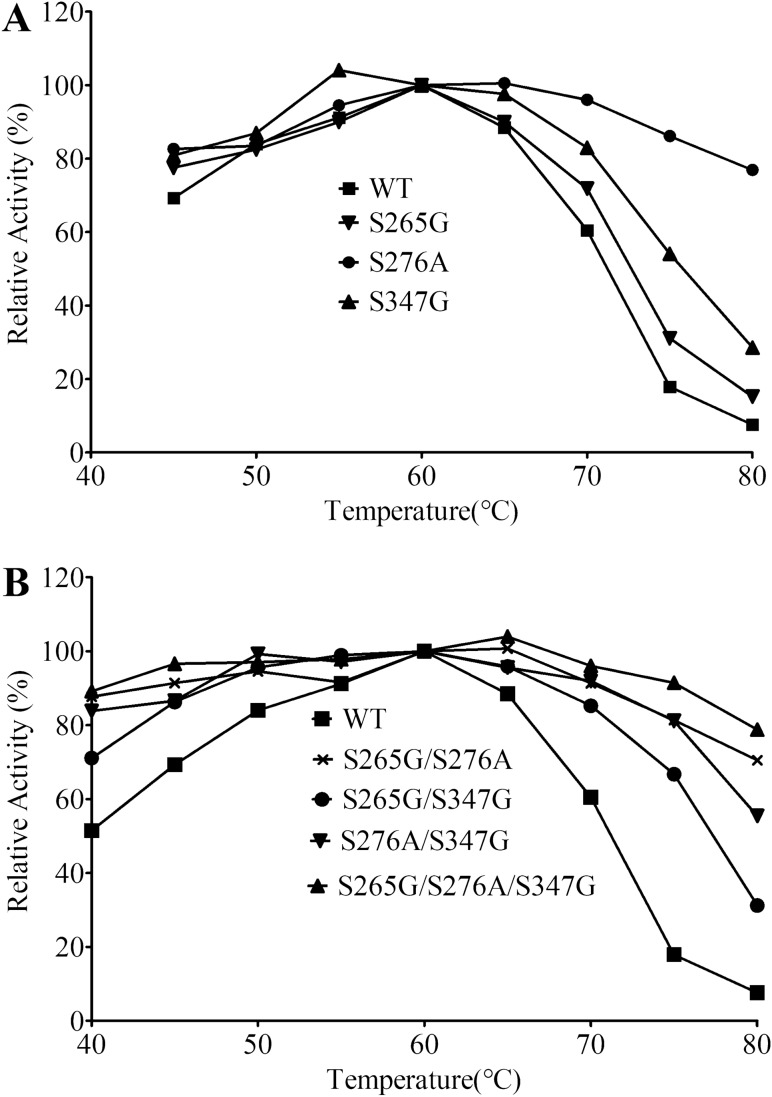
Temperature profiles of the wild-type enzyme and mutant variants. (**A**) The wild-type and single-site mutants. (**B**) the wild-type and multiple-sites mutants.

The CsnTS variants were purified by Ni^2+^-chelating affinity columns and determined by SDS-PAGE. All variants as well as the wild-type CsnTS have a molecular mass of about 46 kDa. The half-lives of the wild-type CsnTS and its variants were measured at 60 °C. As shown in Table 2, the half-life of the wild-type CsnTS was 5.32 min while that of the three mutants S265G, S276A and S347G displayed longer half-lives of 34.57 min, 36.79 min and 7.2 min, respectively. Temperature profiles of the wild-type and all single-site mutants are shown in Fig. 3a. All mutants display a wider range of temperature stability than the wild-type CsnTS. Notably, the mutant S276A exhibits an enzymatic activity of 70%, even at a high temperature of 80 °C while the wild-type enzyme has completely lost its activity at this temperature. The optimal temperature of S347G is 55 °C, which is lower than that of the wild-type enzyme. To study the relationship between the thermal stability and protein conformation, a DSC assay was applied to measure the *T*_*m*_ values of the wild-type and all mutated enzymes. The results are shown in Table 2 and Figure S1. The *T*_*m*_ values of mutants S265G and S276A are increased by 3 °C and 2 °C, respectively, as compared to the *T*_*m*_ of 62 °C of the wild-type enzyme, while S347G displays the decreased *T*_*m*_ value. The optimal pH values of the mutated enzymes are identical to that of the wild-type CsnTS (data not shown).

**Table 2:** Activity and thermostability of csnTS and its mutants: specific activity, *t*_1/2_ (60 °C) and optimum temperature.

Enzyme	Specific activity (U/mg)	*t*_1/2_ (60 °C) (min)	*T*_m_ (°C)
Wild type	566 ± 24	5.32 ± 0.83	62
S265G	279 ± 17	34.57 ± 0.92	65.1
S276A	456 ± 31	36.79 ± 1.01	64.8
S347G	602 ± 25	7.2 ± 0.63	60.4
S265G/S276A	419 ± 17	51.84 ± 0.9	69.3
S265G/S347G	387 ± 15	34.62 ± 1.06	63.1
S276A/S347G	569 ± 45	44.88 ± 0.86	65.5
S265G/S276A/S347G	512 ± 19	55.31 ± 0.88	69.5

The specific activities of mutants S276A and S347G are 456 and 602 U/mg and thus similar to the specific activity of wild-type CsnTS of 566 U/mg, whereas the specific activity of mutant S265G is 279 U/mg and hence significantly lower. The *K*_m_ values of all three mutants are slightly increased. The *K*_cat_ of mutant S276A is increased while the *K*_cat_ of mutant S265G is significantly decreased. Lastly, the S347G mutant has a *K*_cat_ value similar to that of wild-type CsnTS (Table 3).

**Table 3:** Kinetic parameters of wild-type and mutated csnTS.

Enzyme	*K*_m_ (g/l)	*k*_cat_ (10^5^ s^−1^)	*k*_cat_/*K*_m_ (10^5^ l/g·s)
Wild type	1.09 ± 0.09	5.52	5.04
S265G	1.41 ± 0.22	2.4	1.7
S276A	1.32 ± 0.21	8.43	6.37
S347G	1.28 ± 0.19	5.96	4.65
S265G/S276A	1.49 ± 0.17	6.84	4.59
S265G/S347G	1.11 ± 0.1	5.79	5.22
S276A/S347G	1.21 ± 0.12	6.37	5.27
S265G/S276A/S347G	1.16 ± 0.14	6.09	5.24

### Combinations of beneficial mutations

Recent studies have shown that combining two or more beneficial mutations can further improve the thermostability of proteins^7,10,28^. Therefore, we constructed mutant combinations from the identified three mutants to further improve the thermostability of CsnTS.

Our experimental results are as follows: The half-lives of the purified combined mutants S265G/S276A, S265G/S347G, S276A/S347G and S265G/S276A/S347G are 51.84 min, 34.62 min, 44.88 min and 55.31 min at 60 °C (Table 2), respectively. Except for the S265G/S347G mutant, all other combined mutants display a significantly higher thermostability than those of the single mutants. The *T*_m_ values of these combined mutants are about 6 °C higher than the *T*_m_ of wild-type CsnTS. In addition, all combined mutants display stability over a much wider temperature range (Fig. 3b); they all remain enzymatically active on a 80% level for temperatures between 45 and 75 °C. Remarkably, mutant S276A/S347G performed best by exhibiting enzymatic activity between 40 and 80 °C, with an optimal temperature between 50 and 65 °C.

Moreover, the optimal pH values and pH stabilities of the double and triple mutated enzymes are similar to those of wild-type CsnTS and single-site mutants (data not shown). Although the S265G single-site mutant has a significantly reduced enzymatic activity, all other multi-site mutants that include S265G display catalytic abilities similar to that of wild-type CsnTS.

We anticipate our thermostable mutations will become very useful for industrial applications, and may also be further improved by using a methodology similar as presented herein.

### Structural interpretation for increased thermostability in mutants

**Figure 4:**
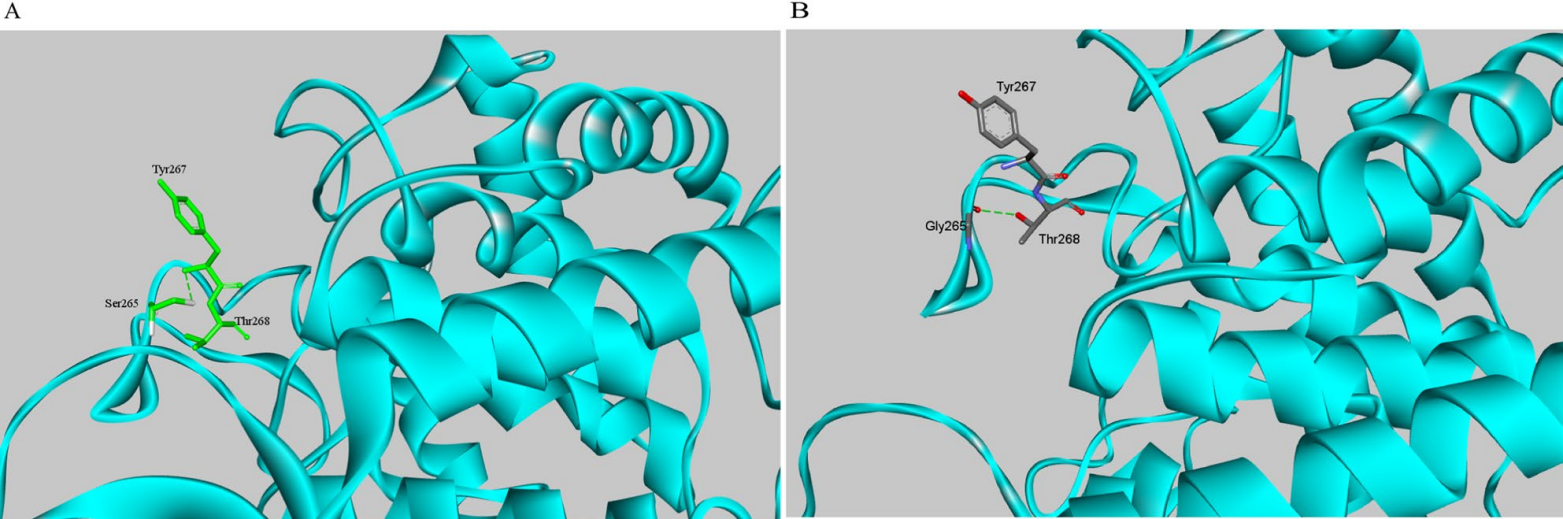
Changes of intramolecular interactions of residue 265 with the nearby residues after S265G mutation. The intramolecular interactions were displayed with Discovery Studio 4.1. The proteins were shown in cyan. The residue Ser265 is shown in green and Gly265 is shown in gray according to the stick representation scheme. The dotted line corresponds to the intramolecular interactions. (**A**) Wild-type. (**B**) S265G.

To better understand the factors that affect the thermostability of these mutants, we compared the generated structures of CsnTS and mutants. As has been previously confirmed, Ser265 forms hydrogen bonds with nearby residues in the endoglucanase CelA^23^. With Gly replacing Ser265, the mutated enzyme has now a half-life of 34.57, which is ~ 7 times longer than the half-life of wild-type chitosanase. On the other hand, the specific activity and catalytic ability of the S265G mutant decreased compared to that of the wild type. Ser265 is on the flexible loop adjacent to the substrate binding position (Fig. 1). Intramolecular interaction analysis revealed that the interaction between S265 and Y267 in wild-type CsnTS is replaced by the interaction between G265 and T268 in the S265G mutant (Fig. 4, Table S1). This change in interactions may stabilize the loop. Moreover, the number of intramolecular interactions increased in the S265G mutant as compared to that in the wild-type enzyme. These interactions reduce the flexibility of the loop, thus improving the mutant’s thermostability while resulting in a loss of its catalytic ability. This is in line with previous observations where site-directed mutagenesis of proteins failed to simultaneously improve thermostability and enzymatic activity^29,30^.

**Figure 5:**
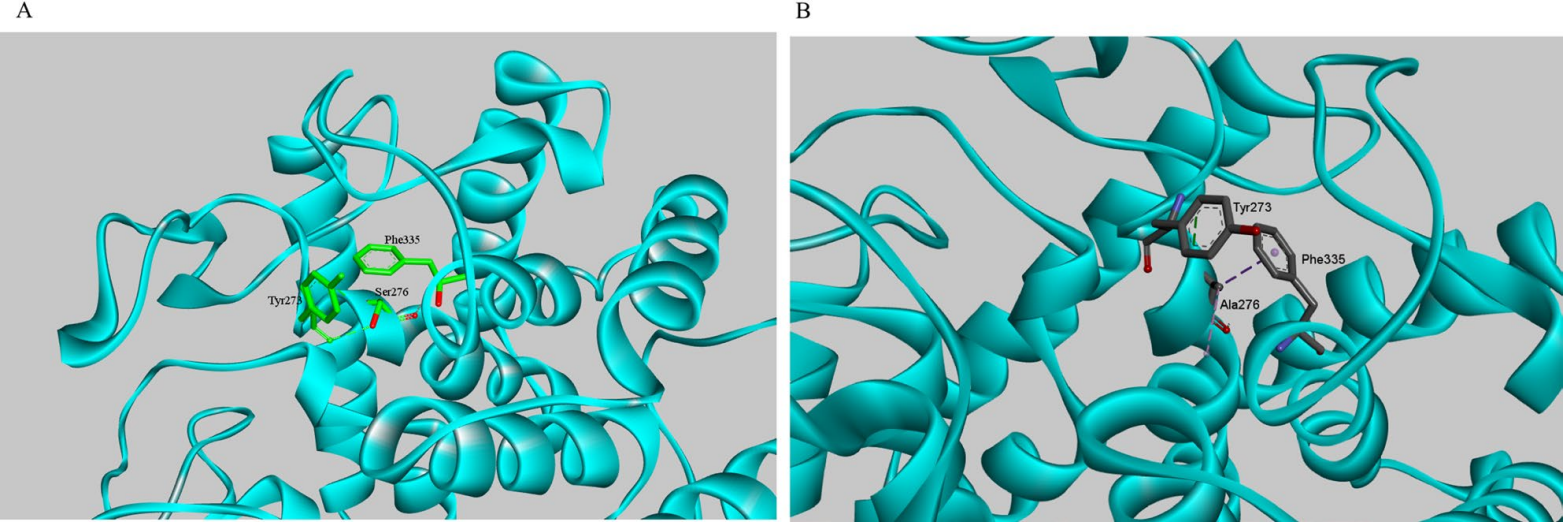
Changes of intramolecular interactions of residue 276 with the nearby residues after S276A mutation. The intramolecular interactions were displayed with Discovery Studio 4.1. The proteins are shown in cyan. The residue Ser276 is shown in green and Ala276 is shown in gray according to the stick representation scheme. The dotted line corresponds to the intramolecular interactions. (**A**) Wild-type. (**B**) S276A.

The Ala276–Phe335 interaction in the S276A mutant substitutes the Ser276–Tyr273 interaction in the wild-type chitosanase (Fig. 5, Table S3). The Ser/Ala276 residue is on the α-helix adhered to the enzymatic activity site, Tyr273 is on the loop at the end of the same α-helix, and Phe335 is nearby but on another α-helix. The substituted intramolecular interaction stabilizes the tertiary structure of the protein. Additionally, it also has been reported that alanine residues are beneficial for the α-helix stability^31^.

Lastly, our analysis of the S265G/S276A/S347G mutant revealed that its intramolecular interactions show many re-arrangements (Table S4). Notably, far more interactions disappeared as compared with those that were created because of the mutation. This could indicate that proper structure arrangements rather than individual intramolecular interactions stabilize the proteins.

## Discussion

The thermostability of proteins can be determined by many important structural features^32^, including hydrogen bonds^33^, salt bridges^19,34^, aromatic π–π interactions, and cation–π interactions. Thermostability can be improved by a number of methods^6^. One such method compares the sequence of a protein with that of a thermostable and highly homologous counterpart. This can suggest residues that are potentially influencing thermostability and may be modified in the original protein. By engineering and screening of enzymes with mutations at such residues, mutants with improved thermostability have been identified for several enzymes already^14,15,35,36^.

In this study, we successfully adopted this method and engineered CsnTS mutants with improved thermostability. We selected several serines as mutation targets through structure comparison of CsnTS and CelA from *Clostridium thermocellum* and simultaneously considering statistical data revealing that serine residues are not favorable for thermostability^23,24^. By screening a total of 15 mutants with a single mutation in one serine residue, we identified three mutations, namely S265G, S276A and S347G, which are particularly beneficial for our purpose. Our study shows that all mutants, with single-site mutations and with combined mutations, exhibit a higher thermostability than the wild-type chitosanase. Moreover, all combined mutants outperformed the single-site mutants.

Statistical analysis of structural distribution of amino acids between thermophilic and mesophilic proteins revealed that serine was observed at low frequency at the surface^21,22,37^. In single serine mutants, the increased intramolecular interactions were found to be the main factors that could enhance thermal stability^38^. However, the number of intramolecular interactions of the multiple mutant S265G/S276A/S347G, is lower than that of the wild-type CsnTS. Thus, we hypothesize the re-arrangements in intramolecular interactions may contribute most toward the enhanced thermostability of the multiple mutant S265G/S276A/S347G.

In summary, we engineered mutants of chitosanase from *Bacillus* sp. TS following structure comparison with CelA from *Clostridium thermocellum*. Mutants S276A and S347G were identified as those that exhibit a higher thermostability in comparison to the wild-type chitosanase without losing catalytic activity. In addition, most of combinations of these mutations enhanced the thermostability of the enzyme further, making these mutants more enzymatically active during hydrolysis of chitosan than wild-type chitosanase. We presented a promising avenue for rational design and anticipate our recombinant mutants will have great potential for industrial applications.

## Supplementary Information

Supplementary Information.

## Data Availability

The datasets used and/or analysed during the current study available from the corresponding author on reasonable request.
