# Supplementary material for: Improve thermostability of Bacillus sp. TS chitosanase through structure-based alignment
Source: Sci Rep. 2021 Aug 4;11:15846. doi: 10.1038/s41598-021-95369-w (PMC8339078; doi:10.1038/s41598-021-95369-w)

Table S1: The intramolecular interactions in S265G mutant.

Newly formed	Disappeared
A:TRP352:NE1 - A:SER343:OG	A:TYR267:N - A:SER265:OG
A:THR268:OG1 - A:GLY265:O	A:ASN178:CA - A:ASN186:OD1
A:TRP352:NE1 - A:SER343:OG	
A:PRO116:CD - A:GLN114:O	
A:VAL223 - A:LEU208	

Table S2: The intramolecular interactions in S276A mutant.

Newly formed	Disappeared
A:TRP352:NE1 - A:SER343:OG	A:SER276:OG - A:TYR273:O
A:PRO279:CD - A:ALA276:O	A:PRO279:CD - A:SER276:O
A:ALA276:CB - A:PHE335	A:ASN178:CA - A:ASN186:OD1
A:ALA276 - A:VAL334	

Table S3: The intramolecular interactions in S347G mutant.

Newly formed	Disappeared
A:GLN350:N - A:GLY347:O	A:GLN350:N - A:SER347:O
A:TRP352:NE1 - A:SER343:OG	

Table S4: The intramolecular interactions in S265G/S276A/S347G mutant.

Newly formed	Disappeared
A:ARG199:NH2 - A:ASP138:OD2	A:ARG199:NH2 - A:ASP138:OD1
A:ARG277:NH1 - A:ASP138:OD1	A:ARG277:NH1 - A:ASP138:OD2
A:ARG199:NH1 - A:ASP138:OD1	A:ARG199:NH1 - A:ASP138:OD2
A:GLY139:N - A:ASP138:OD2	A:GLY139:N - A:ASP138:OD1
A:ARG184:NH1 - A:ASN252:OD1	A:ARG184:NH2 - A:ASN252:OD1
A:LYS21:NZ - A:GLN13:O	A:ASN31:N - A:GLU28:O
A:LYS44:NZ - A:GLU365:O	A:SER33:N - A:LEU30:O
A:ARG108:NH1 - A:LEU118:O	A:TYR37:N - A:VAL34:O
A:ASN117:ND2 - A:LYS172:O	A:ASN40:N - A:TYR37:O
A:GLN130:NE2 - A:SER52:OG	A:LYS43:N - A:ASN40:O
A:ALA150:N - A:LEU147:O	A:SER51:N - A:ASP125:OD1
A:TYR162:N - A:SER156:OG	A:SER52:OG - A:ASP125:OD1
A:ILE174:N - A:ILE170:O	A:GLY74:N - A:VAL123:O
A:THR180:OG1 - A:ARG184:O	A:GLN79:N - A:SER76:O
A:ASN182:ND2 - A:SER194:O	A:TYR81:N - A:GLY78:O
A:ARG184:NH2 - A:ASN182:OD1	A:MET83:N - A:GLY80:O
A:GLY188:N - A:ASN186:OD1	A:VAL87:N - A:ILE84:O
A:VAL223:N - A:TRP220:O	A:MET89:N - A:THR86:O
A:TYR228:N - A:ASN225:O	A:ILE99:N - A:ALA96:O
A:SER235:N - A:THR232:O	A:GLY102:N - A:ILE99:O
A:THR242:N - A:SER239:O	A:PHE104:N - A:ASP101:O
A:THR268:OG1 - A:GLY265:O	A:LYS105:N - A:GLY102:O
A:ALA276:N - A:TYR272:O	A:THR106:OG1 - A:GLY102:O
A:ARG281:N - A:VAL278:O	A:ARG108:NH1 - A:PRO116:O
A:TYR286:N - A:SER294:OG	A:THR109:N - A:THR106:O
A:LYS300:N - A:ILE297:O	A:LYS111:N - A:ARG108:O
A:ILE305:N - A:SER302:O	A:ASP140:N - A:THR137:O
A:THR345:OG1 - A:ASN346:OD1	A:ASP142:N - A:GLY139:O
A:ASN349:ND2 - A:SER302:OG	A:ILE143:N - A:ASP140:O
A:ARG364:NH1 - A:ASN40:OD1	A:LEU147:N - A:ALA144:O
A:TYR367:N - A:GLU62:OE2	A:LEU149:N - A:SER146:O
A:PRO9:CD - A:GLU6:O	A:HIS151:N - A:LEU148:O
A:PRO116:CD - A:GLN114:O	A:TYR162:N - A:VAL160:O
A:ASN161:CA - A:SER156:O	A:GLU165:N - A:TYR162:O
A:SER177:CB - A:ASN178:OD1	A:GLY173:N - A:ASP168:O
A:ARG199:CA - A:ASP247:O	A:SER183:OG - A:ASN182:O
A:SER235:CB - A:TYR231:O	A:ARG184:NH1 - A:LYS251:O
A:GLU266:CA - A:ASP260:OD2	A:LEU187:N - A:ASP191:OD2
A:PRO279:CD - A:ALA276:O	A:THR222:OG1 - A:LYS218:O
A:LYS361:CE - A:SER33:OG	A:ILE224:N - A:LEU221:O
A:ASP191:OD2 - A:TRP190	A:LEU227:N - A:ILE224:O
A:TYR271:OH - A:PHE339	A:VAL230:N - A:LEU227:O

A:ILE84:CG2 - A:TRP384	A:GLN233:N - A:VAL230:O
A:LEU88:CD1 - A:TRP384	A:PHE234:N - A:TYR231:O
A:ALA276:CB - A:PHE335	A:SER235:OG - A:SER239:O
A:ILE84 - A:LEU375	A:SER235:OG - A:SER239:OG
A:LEU88 - A:LEU149	A:SER239:OG - A:SER235:O
A:ALA124 - A:VAL122	A:TYR267:N - A:SER265:OG
A:VAL223 - A:LEU208	A:TYR272:N - A:ALA270:O
A:ALA276 - A:VAL334	A:SER276:N - A:TYR272:O
A:PRO279 - A:VAL301	A:SER276:OG - A:TYR273:O
A:PHE133 - A:ALA129	A:LEU280:N - A:ARG277:O
A:TRP359 - A:PRO331	A:ARG281:N - A:VAL278:O
A:TYR372 - A:PRO338	A:ARG281:NE - A:TYR372:OH
A:TRP384 - A:LEU375	A:GLY290:N - A:ASP285:O
	A:SER298:OG - A:SER294:O
	A:SER302:OG - A:SER343:OG
	A:SER303:OG - A:ASP299:O
	A:GLN306:N - A:SER303:O
	A:ASN310:N - A:ASN307:O
	A:VAL336:N - A:GLY333:O
	A:ILE344:N - A:ALA341:O
	A:ASN346:ND2 - A:ASP299:OD1
	A:ASN346:ND2 - A:THR345:OG1
	A:GLN350:N - A:SER347:O
	A:ASN354:N - A:LYS351:O
	A:SER355:N - A:TRP352:O
	A:ASP358:N - A:SER355:O
	A:TRP359:N - A:GLY356:O
	A:LEU374:N - A:SER371:O
	A:MET377:N - A:LEU374:O
	A:LEU378:N - A:LEU375:O
	A:THR381:OG1 - A:LEU378:O
	A:TYR57:CA - A:LYS47:O
	A:TRP121:CA - A:SER76:OG
	A:SER135:CB - A:ASP140:OD2
	A:ASN178:CA - A:ASN186:OD1
	A:SER201:CB - A:ASN274:O
	A:SER246:CB - A:THR268:O
	A:PRO256:CA - A:SER246:OG
	A:PRO279:CD - A:SER276:O
	A:VAL301:CA - A:THR242:O
	A:GLY319:CA - A:TYR271:O
	A:GLY328:CA - A:ASP318:O
	A:PRO387:CD - A:TRP385:O
	A:PRO389:CD - A:GLU213:OE1

A:ASP191:OD1 - A:TRP190
A:VAL336:CG1 - A:TRP359
A:VAL25 - A:LEU30
A:TRP154 - A:ALA96
A:TRP384 - A:LEU88
A:TRP384 - A:ILE84

Figure S1: DSC curves of the wild-type and mutated chitosanases

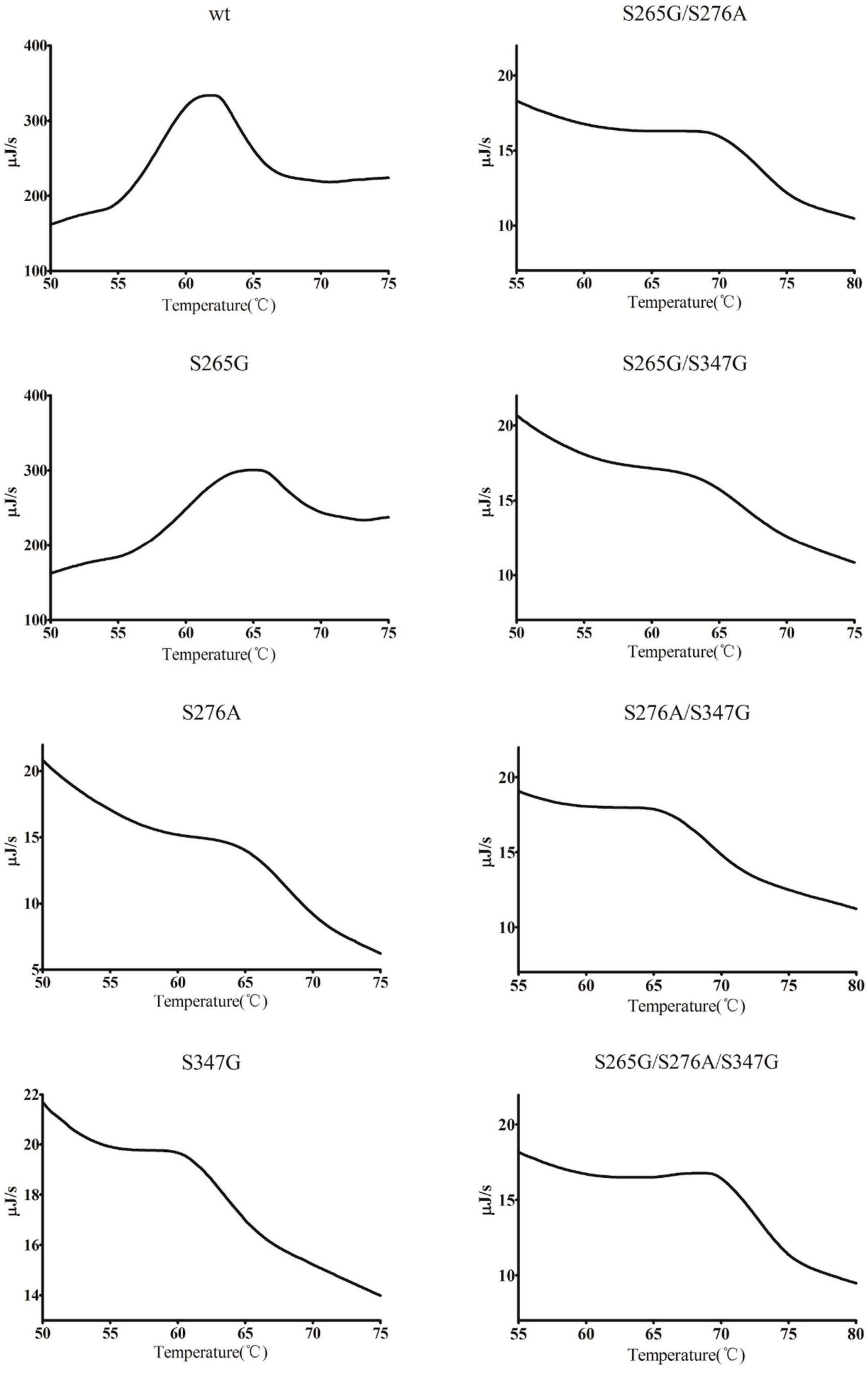

Supplement: Supplementary file 1 — Supplementary Information. [file 41598_2021_95369_MOESM1_ESM.pdf]
